# Optimisation of monolithic nanocomposite and transparent ceramic scintillation detectors for positron emission tomography

**DOI:** 10.1038/s41598-020-58208-y

**Published:** 2020-01-29

**Authors:** Keenan J. Wilson, Roumani Alabd, Mehran Abolhasan, Mitra Safavi-Naeini, Daniel R. Franklin

**Affiliations:** 1 School of Electrical and Data Engineering, University of Technology Sydney, Sydney, NSW Australia; 2 Australian Nuclear Science and Technology Organisation (ANSTO), Sydney, NSW Australia

**Keywords:** Nanoparticles, Imaging techniques, Molecular imaging

## Abstract

High-resolution arrays of discrete monocrystalline scintillators used for gamma photon coincidence detection in PET are costly and complex to fabricate, and exhibit intrinsically non-uniform sensitivity with respect to emission angle. Nanocomposites and transparent ceramics are two alternative classes of scintillator materials which can be formed into large monolithic structures, and which, when coupled to optical photodetector arrays, may offer a pathway to low cost, high-sensitivity, high-resolution PET. However, due to their high optical attenuation and scattering relative to monocrystalline scintillators, these materials exhibit an inherent trade-off between detection sensitivity and the number of scintillation photons which reach the optical photodetectors. In this work, a method for optimising scintillator thickness to maximise the probability of locating the point of interaction of 511 keV photons in a monolithic scintillator within a specified error bound is proposed and evaluated for five nanocomposite materials (LaBr_3_:Ce-polystyrene, Gd_2_O_3_-polyvinyl toluene, LaF_3_:Ce-polystyrene, LaF_3_:Ce-oleic acid and YAG:Ce-polystyrene) and four ceramics (GAGG:Ce, GLuGAG:Ce, GYGAG:Ce and LuAG:Pr). LaF_3_:Ce-polystyrene and GLuGAG:Ce were the best-performing nanocomposite and ceramic materials, respectively, with maximum sensitivities of 48.8% and 67.8% for 5 mm localisation accuracy with scintillator thicknesses of 42.6 mm and 27.5 mm, respectively.

## Introduction

Detection of high-energy photons for positron emission tomography (PET) is inherently challenging due to their highly penetrating nature. Solid-state detectors are unable to directly detect such photons with high efficiency, due to the limited detector thickness (typically <1 mm) and low density and effective atomic number of most semiconductors^1,2^. To increase sensitivity to high energy photons, in most applications it is necessary to optically couple a photodetector to a *scintillator*. When a high-energy photon deposits energy in the scintillator, the energy is absorbed by dopant atoms (commonly cerium, europium or thallium) and re-radiated as multiple lower-energy photons - typically in the optical range - which can be detected by a solid-state photodetector or photomultiplier tube^3^. Many excellent scintillator materials are now available, including some with good sensitivity to high energy photons due to their high density and effective atomic number^3,4^. The best of these also provide high light output, good energy resolution and fast decay time, and scintillate at wavelengths which are compatible with semiconductor photodetectors^3–5^.

Conventionally, high spatial resolution PET requires the use of a large number of very small, discrete, optically isolated scintillator crystals, either individually coupled to a photodetector cell or multiplexed via some sort of light-sharing scheme. High gamma photon sensitivity requires a large radial scintillator thickness, while maintaining a uniformly high spatial resolution across the field of view requires very narrow crystal columns with the ability to determine the depth of interaction (DOI) within the scintillator^6,7^. Each of these requirements contributes to high system cost and manufacturing complexity^8^; reducing crystal dimensions also negatively impacts timing and energy resolution^8,9^ and greatly complicates interfacing the scintillator with optical photodetectors. An alternative to discrete crystals is for each detector block to use a large monolithic scintillator slab coupled to a pixellated photodetector array on one or more of its surfaces, addressing the tradeoff in sensitivity, timing resolution and energy resolution associated with the use of discrete crystals^9–13^. The optical photon distribution arriving at the detector arrays can then be used to estimate the point of interaction within the slab^14–17^. This complicates the task of coincidence detection, but offers the major advantage of simplified manufacturing compared to the discrete-crystal approach. However, growing large crystals of uniformly high quality remains technically challenging and expensive, and machining monocrystalline scintillators into non-rectangular shapes is difficult for most materials^18^.

Two new classes of materials have been proposed as lower-cost alternatives to monocrystalline scintillators, particularly where complex shaping of the scintillator is required: nanocomposites^19^ and transparent ceramics^20^. Nanocomposites are a uniform mixture of inorganic scintillator nanocrystals and a polymer binder (matrix). Although nanocomposites have lower density and effective atomic number compared to the macroscopic form of its inorganic scintillator component, these parameters are still higher than those of pure polymer scintillators^21^. Optical photon yield and emission wavelength are also similar (but not identical) to those of the macroscopic scintillator^21^. Nanocomposites may be handled much like a simple polymer, enabling convenient casting and machining^22^. Transparent ceramic scintillators are chemically related to existing single-crystal scintillator materials, but with a polycrystalline structure. Many of the desirable properties of comparable monocrystalline scintillators are retained (in particular, high density, *Z*_*eff*_ and optical photon yield), however, they can be fabricated more rapidly at much lower temperatures, and formed into complex shapes much more easily than monocrystalline scintillators^23^.

A common shortcoming of both nanocomposites and transparent ceramic scintillators is their lower optical transparency compared to single-crystal scintillators^23–26^. In the case of nanocomposites, the difference between the refractive indices of the nanocrystal scintillator and the matrix polymer leads to Rayleigh scattering and reduced transmittivity^21,23^. A similar problem also exists to some extent in ceramic scintillators due to the presence of grain boundaries in the material. Consequently, with increased monolithic slab thickness, scattering and attenuation in nanocomposite and ceramic scintillators degrade the optical signal to the point where it is no longer useful in providing an accurate estimate of the point of interaction within the scintillator^9,27^. This is quite unlike the situation with monocrystalline materials, which tyipcally exhibit very low optical attenuation and scattering, such that for any practical range of monolithic slab thicknesses these non-idealities can be neglected^28,29^. Therefore, determining the optimal thickness for nanocomposite and ceramic scintillators is an important design question for those wishing to utilise these materials in PET systems and other radiation detection applications. In this context, *optimal* refers to that thickness which maximises the number of photons whose point of interaction can be estimated within a specified threshold of accuracy. Too thin, and the probability of detection will be too low; too thick and many of the optical photons may be absorbed before reaching the optical photodetector, resulting in an inaccurate position estimation.

This work proposes a general method for determining optimal thickness of a monolithic scintillator slab, given the physical properties of the scintillator material, based on cubic spline interpolation between results obtained from a set of Monte Carlo simulations. The method is demonstrated by applying it to a range of nanocomposite and transparent ceramic scintillators. Section 2 presents a review of the literature related to this work - in particular, introducing the nanocomposite and transparent ceramic scintillator materials which are to be compared, and discussing methods for photon localisation in monolithic scintillator slabs. Section 3 introduces the proposed thickness-optimisation technique and describes the simulation parameters used in this work; Section 4 presents results comparing scintillator performance with two fixed thicknesses plus the optimal thickness for each material, and discusses the key implications of these results. Finally, the outcomes of this research are summarised in Section 5.

## Related Work

### Nanocomposite scintillators

Nanocomposite scintillators consist of a mixture of nanometre-scale inorganic scintillating particles, uniformly mixed with a transparent polymer or other organic matrix. The matrix can either be non-scintillating, such as oleic acid (OA), or a polymer scintillator, such as polystyrene (PS) or polyvinyltoluene (PVT)^23^. Because the gaps between scintillator nanoparticles are filled with a material with a refractive index closer to that of the scintillator than air (or vacuum), scattering is limited and light transmission is improved compared to the use of a compressed bulk powder^19^. Nevertheless, no matrix material has a refractive index which perfectly matches that of the nanocrystal; therefore, scattering remains a performance-limiting factor, particularly in comparison to monocrystalline scintillator materials. For hygroscopic materials, the matrix offers the additional advantage of protecting the nanoparticles against moisture ingress.

Accurately modelling the optical properties of a nanocomposite is considerably more complex than for a homogeneous material. Most useful nanoscintillator particles are sufficiently small that scattering is purely Rayleigh (i.e., satisfying the criteria $m\cdot x < 1$, where $m={n}_{p}/{n}_{m}$ is the ratio of the refractive index of the scintillator particles to that of the matrix, and $x=2\pi r/\lambda$ and $\lambda ={\lambda }_{0}/{n}_{m}$ where *λ*_0_ is the peak emission wavelength of the scintillator^30^). If this condition is satisfied, then the transmission of scintillation photons may then be calculated via (1) ^23–26^:

1 $$T=\frac{I}{{I}_{0}}=exp\left\{-4{\pi }^{4}\frac{{d}^{3}\bar{L}{f}_{p}{n}_{m}^{4}}{{\lambda }^{4}}{(\frac{{m}^{2}-1}{{m}^{2}+2})}^{2}\right\}$$

where *I*/*I*_0_ is the ratio of the output light intensity (*I*) to the emission light intensity (*I*_0_), *d* is the diameter of the nanoparticle, *f*_*p*_ is the volume fraction of particles, $\bar{L}$ is the average path length and *λ* is the wavelength of the scintillation photons. This expression assumes that the diameter of nanoparticles is uniform, there is no agglomeration of particles in the matrix, also that the photons emitted during scintillation are of exactly the same wavelength.

In practice, none of these assumptions is strictly accurate. Particle agglomeration degrades uniformity when the nanocomposite loading factor is high, creating additional scattering losses^31^. If there is some overlap between the absorption spectrum of the matrix and the luminescence spectrum of the nanoscintillator, self-absorption of scintillation photons may occur; some polymer matrix materials are known to exhibit this behaviour, especially in the UV region^26,32^. Despite these limitations, (1) provides a good approximation of optical transmission of scintillation photons for the majority of nanocomposite materials.

The refractive index of the nanocomposite (*n*_*c*_) is a function of the nanoparticle loading factor - in fact, much research has been conducted into the problem of using inorganic nanoparticles to effectively tune the refractive index of an organic material. For many nanocomposite scintillators, an *effective medium* model based on Maxwell-Garnett theory can be used to estimate the composite refractive index using an additive linear approximation, as shown in (2) ^33–35^:

2 $${n}_{c}={n}_{m}{f}_{m}+{n}_{p}{f}_{p}$$

where *n*_*m*_ and *n*_*p*_ are the refractive indices of the matrix and nanoparticles, respectively, and *f*_*m*_ and *f*_*p*_ represent the fractions of each material by volume. However, while a large number of nanocomposite scintillator materials are known to follow this linear relationship between volume fraction and overall refractive index, others do not. Instead, it is often necessary to employ empirical methods. For instance, a TiO_2_-polystyrene nanocomposite synthesised by Rao *et al*.^36^ is best described by a quadratic equation. The authors hypothesise that strong chemical bonding between the two materials changed the polarisability of the polymer, resulting in the observed behaviour. Other factors which can impact upon the refractive index include the diameter of the nanoparticles; for PbS-gelatine, there was a significant drop in refractive index compared to the bulk material when the mean diameter of the nanoparticle component was less than 25 nm^35^. Also, if a surfactant coating with a lower refractive index than the nanoparticle is applied to improve the uniformity of the nanocomposite distribution, it may significantly contribute to the overall volume^37^ and hence alter the refractive index.

Using (1) and (2), it is possible to identify the desirable properties for suitable nanoscintillator and matrix materials. These include closely matching scintillator and matrix refractive indices, small nanoparticle size (<10 nm), long emission wavelength and non-overlapping emission and absorption spectra, each of which will help to maximise transmission of the scintillation light^23,38^. For most imaging applications, high light yield per MeV of gamma photon energy, good energy resolution, and a short time constant are also desirable^9^. In addition, the nanocomposite needs to exhibit a high linear attenuation coefficient so as to maximise its sensitivity to high-energy gamma radiation. This requires the nanocomposite to use a high-*ρ*, high-*Z*_*eff*_ nanoscintillator material at a high loading factor. Unfortunately, a high loading factor also reduces the optical transmittivity of the nanocomposite. Supplementary Section 1 describes a heuristic approach to choosing a nanocomposite loading factor which balances the attenuation of the material with its optical transmittivity (see Supplementary Figs. 1 and 2).

In this work, several recent nanocomposites which may be feasible for fabrication into thick monolithic slabs are compared, with a particular focus on materials with a high nanoparticle loading factor. The material properties are summarised in Table 1^23,26,39–42^. In all nanocomposites with the exception of Gd_2_O_3_-PVT, scintillation occurs directly within the nanoparticle. In the case of Gd_2_O_3_-PVT, the optical photons are emitted via a two-step process which involves the creation of excitons on the nanoparticle surface, which subsequently transfer energy to the surrounding PVT matrix; the PVT matrix then transfers energy to a fluor compound via fluorescence resonance energy transfer (FRET)^26^. Both interactions are extremely short-range (of the order of a few nanometres) and should not significantly impact the use of the material as a monolithic scintillator^26,43^.

**Table 1:** Physical and optical properties of a range of proposed nanocomposite materials.

Nanoparticle Matrix	LaBr_3_:Ce PS	Gd_2_O_3_ PVT	LaF_3_:Ce OA	LaF_3_:Ce PS	YAG:Ce PS
Load (% Vol.)	19	4.6	34	50	50
Peak *λ* (nm)	380^+^	550	334	334	550^+^
Yield (ph/keV)	63^+^	22	4.5^+^	4.5^+^	20.3^+^
*R* (% @662 keV)	2.6^+^	11.4	16^+^	16^+^	11.1^+^
Decay (ns)	16^+^	17	30^+^	30^+^	87.9^+^
*ρ* (g/cm^3^)	1.81^*^	1.34^*^	2.59^*^	3.47^*^	2.81^*^
*n* (@peak *λ*)	1.69^*^	1.56^*^	1.52^*^	1.65^*^	1.72^*^
*α* (cm^−1^)	2.00^*^	0.09^*^	2.05^*^	0.15^*^	0.95^*^
Refs.	^39,42^	^26,39^	^23,39,40^	^23,39,40^	^39,41^

Most properties listed are found from the literature; several parameters, marked with an*, have been estimated from the volume fractions listed, assuming a 1 cm thick slab with 9 nm diameter nanoparticles. The properties listed with a^+^ were taken from the bulk crystalline equivalent of the nanoparticle. *R* is the energy resolution; *ρ* is the material density; *α* is the optical linear attenuation coefficient at the peak emission wavelength. PS is polystyrene; PVT is polyvinyl toluene; OA is oleic acid.

### Transparent ceramic scintillators

Monocrystalline doped synthetic garnets, such as yttrium aluminium garnet (YAG) and lutetium aluminium garnet (LuAG) have been been used for decades in photonics applications such as optically pumped lasers^44–47^. Monocrystalline synthetic garnet scintillators have also been proposed for use in medical imaging applications such as PET due to their high density, high scintillation yield and good optical transparency^9,48^. Recent progress in fabrication techniques has enabled the synthesis of certain garnets as optically transparent polycrystalline ceramics^49^. Such ceramics retain many of the properties of the equivalent monocrystalline material (in particular, the high density and linear attenuation coefficient), and may offer a higher scintillation yield in some cases^27^. The principal benefit of multicrystalline transparent ceramics is easier and more cost-effective fabrication, and the flexibility of being able to form the precursor powder into arbitrary shapes prior to sintering - a near-impossibility with monocrystalline materials.

The optical and scintillation properties of transparent ceramics are heavily influenced by the specific manufacturing process used. Usually, the ceramic is formed by sintering, whereby a precursor material is calcinated and milled into nanoparticles (∼100–200 nm) before being pressed and sintered at around 1500–1700 °C^50,51^. The resulting solid may then undergo hot isostatic pressing (HIP) to reduce porosity, followed by annealing and polishing to create a highly transparent solid^52,53^. The atmosphere in which the sintering and annealing steps are performed has a significant effect on the transparency of the resulting ceramic, and its propensity to exhibit afterglow effects^54^.

Compared to the Czochralski, Kyropoulos or Bridgman-Stockbarger methods used to grow large single-crystal scintillators, the process of creating transparent ceramic scintillators is much more amenable to the production of complex geometries and scaling up to large volumes^29^. Growth of high-quality defect-free monocrystalline scintillators with consistent characteristics requires extremely high, uniform and well-regulated temperatures and atmospheric conditions^28^. Additionally, the maximum dimensions of monocrystalline scintillators are limited by both the maximum dimensions of the ampoules (in the Bridgeman-Stockbarger method) and by the difficulty in maintaining a stable, uniform thermal gradient across a wide crystallisation zone^55,56^. The ability of the ceramics to be pressed into different molded shapes eliminates most of these problems, and the lower temperatures involved mean that material losses are able to be more easily controlled since there is no risk of evaporation of the melt.

A significant challenge in the production of ceramic scintillators is achieving high optical transparency in the finished product. Cherepy *et al*. proposed a method to reduce the residual porosity of the ceramic with hot isostatic pressing, greatly improving optical transparency^57^. Optical properties are also improved through the selection of high purity precursor materials with cubic/isotropic crystal structures (i.e. no birefringence), which reduces optical scatter caused by grain boundaries and secondary phases^57^.

The properties of several ceramic garnet scintillators are listed in Table 2^51–54,57–60^.

**Table 2:** Properties of several transparent ceramic scintillator materials proposed for radiation detection applications.

Ceramic	GYGAG:Ce	GLuGAG:Ce	GAGG:Ce	LuAG:Pr
Peak *λ* (nm)	550	550	530	310
Yield (ph/keV)	50	48.2	70	21.8
R (% @662 keV)	4.9	7.1	4.9	4.6
st Decay (ns)	100	84	90	21.4
nd Decay (ns)	500	148	194	771
*ρ* (g/cm^3^)	5.8	6.9	6.63	6.73
n (@peak *λ*)	1.82	1.92	1.90^*^	2.03^*^
*α* (cm^−1^)	0.10	2.00	3.13^*^	2.86^*^
refs.	^53,54,57^	^52,58^	^59,60^	^51,60^

Properties listed with ^*^ have been calculated, while those with * were obtained from literature pertaining to the equivalent monocrystalline form of the material. *R* is the energy resolution; *ρ* is the material density; *α* is the optical linear attenuation coefficient at the peak emission wavelength.

### Monolithic scintillator interaction localisation

Several methods have been proposed to estimate the point of interaction between a gamma photon and a monolithic scintillator using the distribution of optical photons exiting one or more surfaces of the monolithic slab^10,15,16,61^. Statistical algorithms such as maximum likelihood (ML)^10,61^, chi-squared (*χ*^2^)^62^ or k-nearest-neighbour kNN^62–64^ have been used to fit the optical photon distribution to a library of reference profiles, and thereby locate the point of interaction. However, generating the reference profiles requires either laborious experimental measurements or extensive Monte Carlo simulations, since a large number of events must be recorded over a range of known positions and angles of incidence - an effort which must be repeated for scintillators with different dimensions or material properties. An alternative approach is to fit an analytic model of the optical photon distribution using an optimisation algorithm which minimises the mean squared error between the observed distribution and the analytic prediction^65,66^. Where an exact analytic model is difficult or impractical to derive due to complex optical properties of the material, neural networks can be trained to automatically account for non-linearities in the relationship between the observed photon distribution and the point of interaction, particularly near slab boundaries^67^. However, the training procedure would need to be repeated if the material or slab dimensions change. Therefore, while the neural-network approach theoretically offers superior performance, in this work, the simpler analytic model fitting approach is adopted since the retraining process can be avoided.

The optical photon distribution which will be observed depends on the nature of the interaction of the gamma photon with the scintillator. For cases where the photon is photoelectrically absorbed, all of the optical photons will be emitted from a single point, with the number of optical photons proportional to the energy of the gamma photon. A similar outcome will result from Compton interactions where the scattered photon escapes from the scintillator slab; in this case, the number of photons emitted at the point of interaction will be proportional to the difference in energy between the incident and scattered photon. These two cases will be the easiest to localise due to the simplicity of the interaction; the scintillation event may be treated as an isotropic point source^66,67^. In a nanocomposite or ceramic garnet scintillator, an attenuative factor is included to account for the imperfect transparency of the material^68^.

Where multiple energy depositions occur within the scintillator (for example, a Compton interaction followed by photoelectric absorption of the scattered photon), the optical photon distribution becomes more complex, and accurately fitting an analytic model to the observed optical photon distribution is much harder. It is therefore expected that these interactions will result in a larger position estimation error compared to pure photoelectric events.

## Materials and Methods

In this section, a Monte Carlo-based approach to the optimisation of monolithic scintillator slab thickness for a given material is described. The method is applicable to any material with accurately characterised physical and optical properties. Simulations are performed using version 8.0 of the Geant4 Application for Tomographic Emission (GATE), which extends the well-known Geant4 Monte Carlo framework to enable complex simulations of the interaction of radiation and matter to be constructed via text-based descriptions of geometry and materials^69–71^.

### Performance metric and optimisation algorithm

The probability of a gamma photon interacting with the scintillator in any way (for 511 keV photons, this is either by photoelectric or Compton scattering) is determined by its thickness and linear attenuation coefficient at the photon wavelength. For imaging systems based on *monolithic* scintillators, detection probability can also be defined more specifically, also taking into account the *accuracy* of detection – the *probability that the location of the first energy-depositing interaction within the scintillator can be estimated to within a certain distance of the true location inside the scintillator*. Using this metric, the optimal thickness of a monolithic scintillator may be defined as *the thickness which enables the greatest fraction of incident photons to be localised to within a specified distance of the true point of interaction*. It is reasonable to expect that such an optimum exists; if the scintillator is too thin, detection efficiency will be very low due to the small number of primary photons which will interact with it (although they can be localised very precisely within the slab). If it is too thick, the optical attenuation of the scintillator will cause too many of the scintillation photons to be scattered or absorbed to allow the location of the point interaction to be accurately determined, and the probability of multiple Compton scatter events (which manifest as a multi-point emission within the scintillator, degrading localisation accuracy) increases.

To determine the optimal monolithic scintillator thickness for each material, for a given gamma photon energy, a series of GATE simulations of a given scintillator material are performed with different slab thicknesses. An exhaustive evaluation may be performed for a range of potential thicknesses; however, for most materials this is not necessary. The optimal thickness can be determined by the following procedure:

1. Choose the desired threshold of detection accuracy suitable for the proposed application;
2. Simulate photon interactions with the scintillator over 5–10 thicknesses;
3. Apply the chosen localisation algorithm to the optical photon maps observed on both sides of the scintillator;
4. Determine the probability of being able to estimate the first point of interaction of the incident gamma photons to within the chosen threshold for each simulation; and
5. Apply cubic spline interpolation between the measured points and find the maximum.

In this work, an error-minimisation method is used to fit the location and emission intensity parameters of an approximate analytical model to the optical photon distributions detected on the front and back surfaces of the scintillator.

### Detector geometry

**Figure 1:**
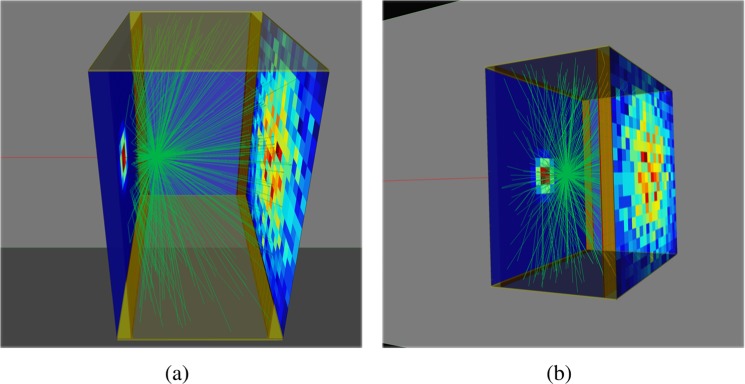
A single photoelectric interaction with a 10 mm thick GYGAG:Ce scintillator slab with front and back photodetectors. The γ photon (red) interacts with the scintillator and emits a shower of 550 nm optical photons (green). Only a small fraction of the optical photon paths are shown for clarity. VRML2 outputs from GATE version 8.0/Geant4 10.2.2 are visualised using view3dscene 3.18.0^77^.

The scintillation detector geometry is similar to the monolithic scintillator/detector model used by Maas *et al*. and simulated in Geant4 and GATE^72,73^. In this study, the lateral dimensions of the scintillator are 20 mm×20 mm, while the thickness is varied; Fig. 1 shows an example of a 10 mm thick GYGAG slab.

The photodetectors are arranged in a double-sided readout (DSR) configuration, which enables accurate localisation of the point of interaction over a wider range of scintillator thicknesses compared to either front-only or back-only readout systems^63^. This is particularly important for the lower density nanocomposite materials. The photodetectors are modelled as a pixellated silicon photodiode; the pixellated detector planes are coupled to the scintillator via thin layers of Meltmount and epoxy (0.05 mm and 0.1 mm respectively), while the exterior surface is covered by a 1 mm polymer/metal layer representing the electronics in the detector. Non-detector faces of the scintillator are covered with non-reflective paint. Detector pixels are 1 mm×1 mm (with zero dead space).

### Monte carlo simulations

To evaluate the chosen performance metric and develop a model for its optimisation, a series of GATE simulations were performed, using the geometry described in Section 3.2. Slab thickness was varied between 5 mm and 35 mm in steps of 5 mm for the transparent ceramics, and between 10 mm and 70 mm in steps of 10 mm for the nanocomposites.

The following parameters were used with GATE version 8.0 and Geant4 version 10.2.2.

#### System

A GATE *optical system* is implemented for accurate optical tracking of the scintillation photons and modelling the relevant physics, including optical attenuation, scattering and surface properties.

#### Physics

The Geant4 low background experiment (LBE) physics list is used, as it is recommended for simulations incorporating optical photon transportation and scintillation processes^74^. Compton scattering and photoelectric processes have been added for gamma photon interactions, along with all processes relating to optical interactions, including bulk absorption, Rayleigh scattering, Mie scattering and boundary processes.

#### Surface properties

Geant4’s UNIFIED optical surface model was used to define the optical properties of the interfaces between materials^75^. To simulate the non-reflective paint covering the non-detector surfaces of the scintillator, the *ground-front-painted* finish was used; reflectivity is set to zero so that optical photons are not internally reflected. The optical roughness of detector-coupled scintillator surfaces has previously been shown to have a minimal influence on localisation accuracy due to the large size of detector pixels relative to the micro-facets^63,76^; therefore, this parameter has been set to ${\sigma }_{\alpha }=0.0$ using the *perfect-apd* model, which assumes a perfectly polished finish and 100% efficiency.

#### Source

The source is a simple monochromatic 511 keV photon gun. Since the focus of this work is providing a mechanism to compare optimal thicknesses of different scintillator materials, rather than the localisation algorithm itself, all photons are directed at the centre of the scintillator slab, parallel to the *z* axis. For optimal thickness determination, 20000 primary (*γ*) photons have been directed at the detector for each evaluated combination of scintillator thickness and material. For determination of spatial error and analysis of scatter composition, more statistical confidence is required; therefore, 200000 primary photons were used. While in a realistic PET scanner, the angle of arrival (AOR) would not, in general, be perpendicular to the scintillator slab, we found that varying the AOR did not significantly impact the accuracy of localisation, which is concerned with identifying the first point of interaction within the scintillator.

### Scintillation event position determination

**Figure 2:**
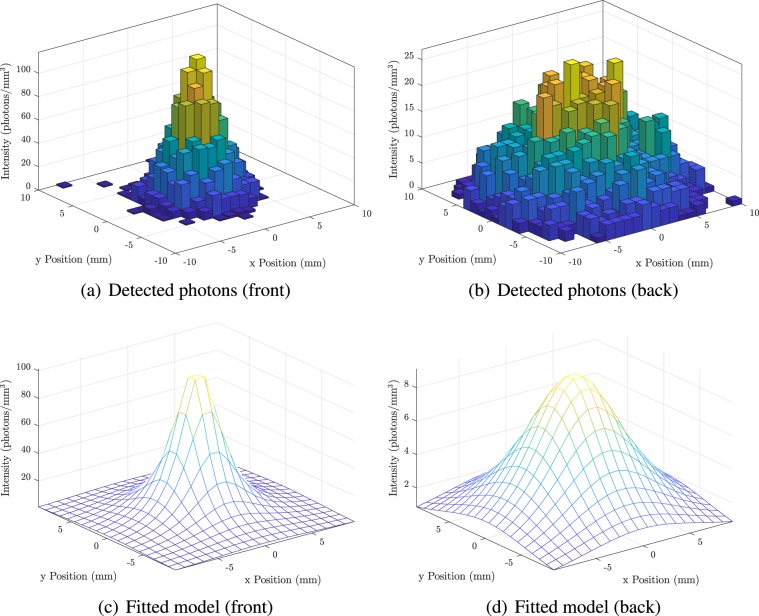
An analytic model of the optical photon distribution is fitted to the observed two-sided photon distribution on the front and back of the scintillator. Example optical photon distributions on the front and back of the detector are shown in (**a**,**b**) respectively, while (**c**,**d**) show the analytic functions fitted to these distributions using (3) and (4). Figures are rendered using Matlab R2019b^78^.

Estimation of the most likely coordinates of the first point of interaction for each (interacting) primary event is performed in Matlab through analysis of the “Hits” list-mode data recorded during the simulation. This file records the location of all photon interactions with the scintillator and detector (both gamma and optical photons). The true 3D points of interaction with the scintillator, as well as the number of photoelectric, Compton and Rayleigh scatter events which occur inside the scintillator material for each interacting primary (*γ*) photon are logged, together the 3D coordinates at which any scintillation photons interact with the detector surface. The time of each interaction and deposited energy are also logged. An example of the detected photon distributions is shown in Fig. 2(a,b).

Detected optical photons are isolated into those associated with an individual primary via their event ID. The optical photon distributions on the surface of each detector resulting are binned into 1 mm × 1 mm pixels.

**Figure 3:**
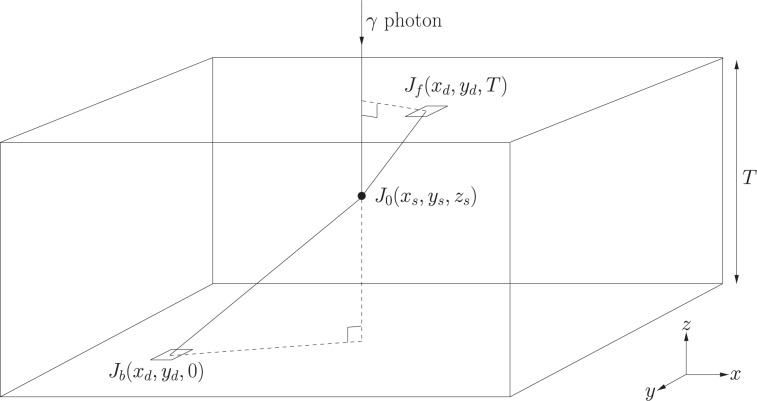
The incident γ photon interacts with the scintillator slab at point $({x}_{s},{y}_{s},{z}_{s})$, resulting in the emission of *J*_0_ optical photons. The number of optical photons arriving at each pixel of dimensions $\Delta x\,\times \,\Delta y$ at position $({x}_{d},{y}_{d})$ on the back and front faces of the detector are given by *J*_*b*_ (3) and *J*_*f*_ (4) respectively. Figure prepared using XFig 3.2.7b^79^.

A parametric model of the predicted photon distribution (as a function of location within the scintillator slab) is fitted to the discrete photon map via the Levenberg-Marquardt minimisation algorithm. The geometry is described in Fig. 3. For scintillation occurring at (*x*_*s*_, *y*_*s*_, *z*_*s*_), the number of photons detected by a pixel of dimensions (Δ*x* × Δ*y*) located at (*x*_*d*_, *y*_*d*_) on the back face is given by:

3 $${J}_{b}({x}_{d},{y}_{d})=\frac{{J}_{0}{z}_{p}\Delta x\Delta y{e}^{-\frac{\sqrt{{({x}_{s}-{x}_{d})}^{2}+{({y}_{s}-{y}_{d})}^{2}+{z}_{s}^{2}}}{\lambda }}}{4\pi {({({x}_{s}-{x}_{d})}^{2}+{({y}_{s}-{y}_{d})}^{2}+{z}_{s}^{2})}^{\frac{3}{2}}}$$

where *J*_0_ is the number of emitted scintillation photons, and *λ* is the radiation attenuation length. Similarly, on the front face (i.e. the first face through which the incident photon passes), the expected number of photons arriving at the (*x*_*d*_, *y*_*d*_) is given by:

4 $${J}_{f}({x}_{d},{y}_{d})=\frac{{J}_{0}(T-{z}_{p})\Delta x\Delta y{e}^{-\frac{\sqrt{{({x}_{s}-{x}_{d})}^{2}+{({y}_{s}-{y}_{d})}^{2}+{(T-{z}_{s})}^{2}}}{\lambda }}}{4\pi {({({x}_{s}-{x}_{d})}^{2}+{({y}_{s}-{y}_{d})}^{2}+{(T-{z}_{s})}^{2})}^{\frac{3}{2}}}$$

where *T* is the thickness of the scintillator slab.

To account for total internal reflection within the slab, *J*_*b*_ = 0 outside of a circle of radius

5 $${R}_{b}={z}_{s}\frac{{n}_{mm}}{{n}_{s}^{2}-{n}_{mm}^{2}}$$

centred at (*x*_*s*_, *y*_*s*_), where *n*_*mm*_ is the refractive index of the optical coupling compound Meltmount (available in the range 1.5 ≤ *n*_*mm*_ ≤ 1.7; in this work it is assumed that *n*_*mm*_ = 1.536) and *n*_*s*_ is the scintillator’s refractive index; if ${R}_{b} < 0$ (which would happen if the refractive index of the scintillator is lower than Meltmount, as would be the case only for LaF_3_:Ce with an oleic acid matrix), there will be no total internal reflection.

Similarly, *J*_*f*_ = 0 outside of a circle of radius.

6 $${R}_{f}=(T-{z}_{s})\frac{{n}_{mm}}{{n}_{s}^{2}-{n}_{mm}^{2}}$$

An example of the fitted analytic model is shown in Fig. 2(c,d).

### Materials

Scintillator properties were taken from the literature where possible (see Tables 1 and 2) or, for the case of the nanocomposites, calculated via the methods described in Section 2 (see (1) and (2)). Several of the optical and physical properties, including the density, refractive index and optical absorption length are dependent upon the loading factor of the nanoscintillator relative to the binder. The scintillation spectrum is assumed to be monochromatic at the peak emission wavelength, so refractive index and absorption length are single values (calculated for the peak wavelength).

The silicon and epoxy layers use the default parameters found in the GATE material database, while MeltmountTM optical coupling compound has been created using the chemical composition described by Van der Laan *et al*.^73^.

As a reference material, equivalent simulations were also conducted using Lu_2_SiO_5_ (LSO), which is a well known scintillation material, common in many PET systems.

## Results and Discussion

### Optimal scintillator thickness

**Figure 4:**
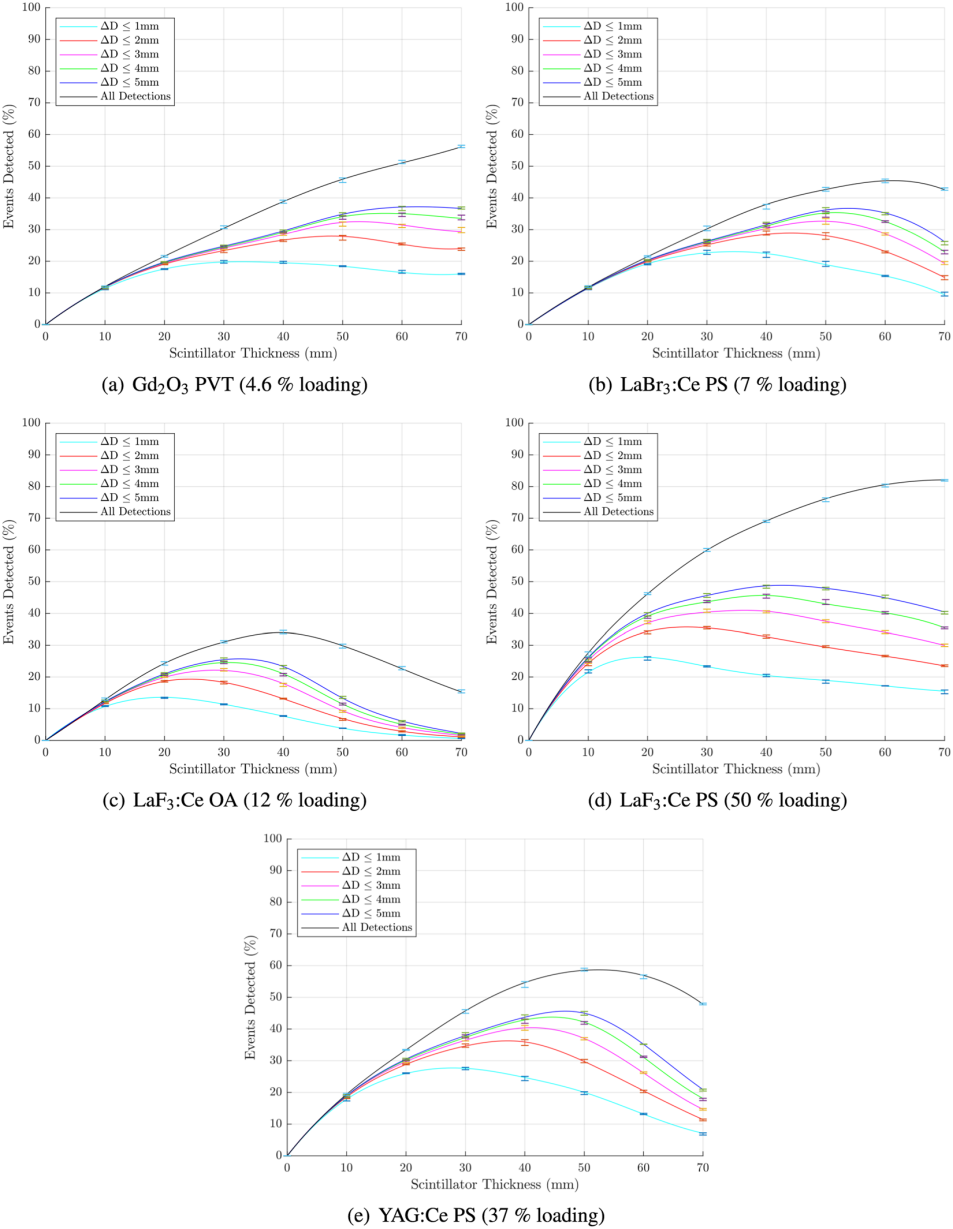
Percentage of events detected to a specified accuracy of 1 mm, 2 mm, 3 mm, 4 mm, 5 mm and all detections as a function of scintillator thicknesses for nanocomposite materials. Median values with interquartile ranges (central 50%) are shown. Figures prepared using Matlab 2019b^78^.

**Figure 5:**
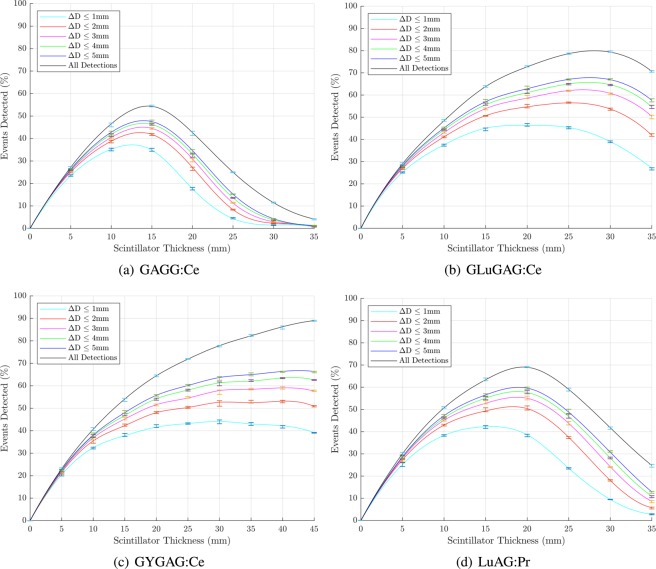
Percentage of events detected to a specified accuracy of 1 mm, 2 mm, 3 mm, 4 mm, 5 mm and all detections as a function of scintillator thicknesses for transparent ceramic materials. Median values with interquartile ranges (central 50%) are shown. Note that the *x*-axis is slightly longer for GYGAG. Figures prepared using Matlab 2019b^78^.

The relationship between scintillator thickness and the number of events detected is shown in Figs. 4 and 5 for the nanocomposite and transparent ceramic scintillators, respectively. These plots include separate curves for detections whose estimated point of interaction has been correctly detected within spatial error limits of 1 mm, 2 mm, 3 mm, 4 mm, 5 mm and all detections regardless off accuracy; Δ*D* is the total spatial error ($\Delta D=\sqrt{\Delta {x}^{2}+\Delta {y}^{2}+\Delta {z}^{2}}$). The location of the maxima of each of these curves is summarised in Table 3.

**Table 3:** Optimum scintillator thickness (denoted T. Opt.) and corresponding probability of detection (P. D.) within a limit of 1, 2, 3, 4, 5 and *∞* mm.

Scintillator	ΔD ≤ 1 mm		ΔD ≤ 2 mm		ΔD ≤ 3 mm		ΔD ≤ 4 mm		ΔD ≤ 5 mm		All Detections	
	T. Opt. (mm)	P.D. (%)	T. Opt. (mm)	P.D. (%)	T. Opt. (mm)	P.D. (%)	T. Opt. (mm)	P.D. (%)	T. Opt. (mm)	P.D. (%)	T. Opt. (mm)	P.D. (%)
Gd_2_O_3_ PVT	32.91	19.84	48.18	27.93	52.71	32.52	57.18	35.15	62.61	37.21	—	—
LaBr_3_:Ce PS	34.56	23.02	44.13	28.88	49.33	32.67	51.61	35.36	53.78	36.70	61.33	45.45
LaF_3_:Ce OA	19.58	13.58	24.26	19.33	28.50	22.15	30.78	24.58	32.66	25.73	39.42	33.97
LaF_3_:Ce PS	19.21	26.23	26.61	35.73	36.35	40.95	39.41	45.66	42.60	48.83	—	—
YAG:Ce PS	27.91	27.72	37.12	36.28	41.13	40.43	44.61	43.76	46.79	45.63	52.43	58.69
GAGG:Ce	12.58	37.17	13.57	42.66	13.85	45.04	13.91	46.73	13.98	47.95	14.53	54.46
GLuGAG:Ce	19.25	46.33	25.25	56.48	26.70	62.37	27.39	65.71	27.54	67.82	28.08	79.94
GYGAG:Ce	29.78	44.13	31.13	52.78	40.54	59.09	41.68	63.67	42.63	66.75	—	—
LuAG:Pr	15.69	42.28	18.31	51.26	18.78	55.50	18.81	58.08	18.96	59.95	19.52	69.09

ΔD is the total error in position estimation for the point of interaction, for both nanocomposite and transparent ceramic scintillator materials.

Additional results for LaBr_3_:Ce PS and LaF_3_:Ce PS with different loading factors are presented in Supplementary Section 2 (Supplementary Figs. 3, 4 and Tables 2, 3).

Of the nanocomposite materials evaluated, LaF_3_:Ce-polystyrene offers the maximum probability of detection for Δ*D* ≥ 3 mm, and is only slightly worse than YAG:Ce-PS at Δ*D* ≤ 2 mm; in all cases, this sensitivity is achieved by LaF_3_:Ce-polystyrene with a much thinner slab of scintillator than for the other materials. This is chiefly due to the high loading factor (and hence high density and *Z*_*eff*_) relative to the other nanocomposite materials. As previously discussed, however, the loading factor cannot be increased without compromising the transmittivity of the nanocomposite. YAG:Ce-polystyrene offers a fair compromise between sensitivity and accuracy of interaction localisation. It has the highest probability of detection (of the nanocomposites) for Δ*D* ≤ 1 mm and Δ*D* ≤ 2 mm and offers sensitivity which is comparable to LaF_3_:Ce-PS for Δ*D* ≤ 3 mm.

With their high density and *Z*_*eff*_, the transparent ceramics offer a much higher probability of detection at any given slab thickness compared to the nanocomposite materials. As with the nanocomposites, the key differences between the different ceramic materials related to detection efficiency and accuracy of position estimation are their emission wavelengths and their optical transmittivity. GAGG:Ce, GLuGAG:Ce and LuAG:Pr all have similar attenuation coefficients at 511 keV, however GYGAG:Ce has by far the highest optical transmittivity, allowing a higher proportion of the the scintillation photons to reach the detectors. While GYGAG:Ce achieves the highest overall probability of detection, the thickness required for this to occur is ≥45 mm and a high proportion of events are localised with an error Δ*D* ≥ 5 mm. As for the nanocomposites, a higher scintillator thickness increases the impact of coherent optical photon scattering, making it increasingly difficult for the localisation algorithm to accurately estimate the point of interaction. The benefits of high optical transparency are therefore limited if density and *Z*_*eff*_ are such that a very thick slab of scintillator is required, since although a large fraction of photons will be detected, their point of interaction cannot be accurately localised.

GLuGAG:Ce provides the highest probability of detection for all finite Δ*D* limits. This is the most dense material while being marginally more transparent than GAGG:Ce and LuAG:Pr. If the scanner is to be optimised for accuracy rather than sensitivity, an optimal thickness for GLuGAG:Ce would be approximately 20 mm, yielding detection of about 46% of photons to within 1 mm of their true point of interaction, and 62.5% of photons within 5 mm. On the other hand, increasing the scintillator thickness to around 27.5 mm will increase the latter figure of merit to 67.5% while reducing the former to around 43% - demonstrating that further increases in scintillator thickness will capture more photons, but with progressively decreasing accuracy. The maximum sensitivity of GAGG:Ce and LuAG:Pr are somewhat lower, peaking at around 48% and 60% of incident photons, respectively (for events localised to within 5 mm of true location).

In summary, while all transparent ceramic scintillator materials significantly outperform the evaluated nanocomposite materials, there may be price points at which certain nanocomposite materials offer sufficient performance to justify their use in a PET scanner design.

### Spatial error

The mean and median errors in each dimension of the estimated positions of gamma photon interactions (where the ground truth is defined as the first point of interaction in the scintillator slab) together with their interquartile ranges (central 50% of errors) and standard deviations are summarised for the optimised-thickness slabs in Table 4 These results include all event types (both pure photoelectric events and events including one or more Compton scatters; the relative probability of different classes of interactions is described in Supplementary Section 3 - Supplementary Tables 4–7). Monocrystalline LSO has again been used as a benchmark scintillator for comparison with each of the materials. Additional results for fixed thicknesses of 1 cm and 2 cm as well as the half-value layer thickness are included in the supplementary material.

**Table 4:** Mean and median errors in the estimation of the point of interaction within a scintillator slab with thickness equal to the calculated optimal thickness, in each dimension and overall.

Scintillator	x error (mm)				y error (mm)				z error (mm)				Total error (mm)			
	Med.	Mean	IQR	SD	Med.	Mean	IQR	SD	Med.	Mean	IQR	SD	Med.	Mean	IQR	SD
Gd_2_O_3_ PVT	$2\times {10}^{-4}$	$7\times {10}^{-3}$	1.0	2.0	$1\times {10}^{-3}$	$6\times {10}^{-3}$	1.0	2.0	$-1\times {10}^{-1}$	0.9	1.2	5.7	1.6	3.0	2.9	5.7
LaBr_3_:Ce PS	$-6\times {10}^{-4}$	$-4\times {10}^{-3}$	0.7	1.6	$4\times {10}^{-4}$	$5\times {10}^{-3}$	0.7	1.6	$-1\times {10}^{-1}$	0.4	1.0	3.7	1.2	2.3	2.3	3.7
LaF_3_:Ce OA	$-2\times {10}^{-3}$	$2\times {10}^{-4}$	1.2	2.1	$9\times {10}^{-4}$	$2\times {10}^{-3}$	1.2	2.1	$-7\times {10}^{-2}$	0.7	1.5	4.2	1.7	3.1	2.7	4.2
LaF_3_:Ce PS	$1\times {10}^{-3}$	$1\times {10}^{-3}$	1.2	2.1	$4\times {10}^{-6}$	$4\times {10}^{-5}$	1.2	2.1	$-7\times {10}^{-2}$	1.1	1.6	5.3	1.8	3.4	3.2	5.1
YAG:Ce PS	$2\times {10}^{-4}$	$6\times {10}^{-3}$	0.8	1.7	$-7\times {10}^{-4}$	$-6\times {10}^{-3}$	0.8	1.7	$-1\times {10}^{-1}$	0.7	1.1	4.5	1.3	2.7	2.4	4.4
GAGG:Ce	$4\times {10}^{-4}$	$2\times {10}^{-3}$	0.3	1.4	$-6\times {10}^{-4}$	$-8\times {10}^{-3}$	0.3	1.4	$-1\times {10}^{-1}$	0.3	0.6	2.0	0.5	1.5	1.1	2.4
GLuGAG:Ce	$-2\times {10}^{-4}$	$-1\times {10}^{-3}$	0.6	1.7	$5\times {10}^{-4}$	$-1\times {10}^{-3}$	0.5	1.7	$-7\times {10}^{-2}$	1.0	1.1	3.6	0.8	2.3	2.0	3.7
GYGAG:Ce	$-1\times {10}^{-4}$	$-4\times {10}^{-3}$	0.7	1.8	$-7\times {10}^{-5}$	$2\times {10}^{-3}$	0.7	1.8	$-8\times {10}^{-2}$	1.5	1.3	5.8	1.0	3.2	2.8	5.7
LuAG:Pr	$9\times {10}^{-4}$	$8\times {10}^{-3}$	0.5	1.5	$7\times {10}^{-4}$	$-9\times {10}^{-5}$	0.5	1.4	$-6\times {10}^{-2}$	0.6	0.9	2.6	0.7	1.9	1.5	2.8

Standard deviations and interquartile ranges (the spread of the middle 50% of errors) are also listed.

The mean and median errors in *x* and *y* dimensions are close to zero for all materials due to the geometric symmetry of the simulation model; the mean errors in *z* range from 0.3 (GAGG:Ce) to 1.5 mm (GYGAG:Ce). The spread spread of error in *x* and *y* is smaller than in *z*, with the standard deviation varying from 1.4 (GAGG:Ce and LuAG:Ce ceramics) to 2.1 (both LaF_3_:Ce OA and LaF_3_:Ce PS nanocomposites); in *z* the standard deviation of the error ranges from 2.0 (GAGG:Ce) to 5.8 (GYGAG:Ce). The difference in error spread is much more pronounced for scintillators with greater optimal thicknesses to two factors: the increased probability of multiple interactions, and increased Rayleigh scattering and attenuation prior to optical photon detection.

Additional localisation error distribution results for fixed scintillator thicknesses of 1 cm and 2 cm are presented in Supplementary Section 4 (Supplementary Tables 8 and 9); a further table of localisation error distributions is provided in Supplementary Section 5 (Supplementary Table 10), where each the thickness of each scintillator slab is the half-value layer (50% gamma photon attenuation thickness) for that particular material.

**Figure 6:**
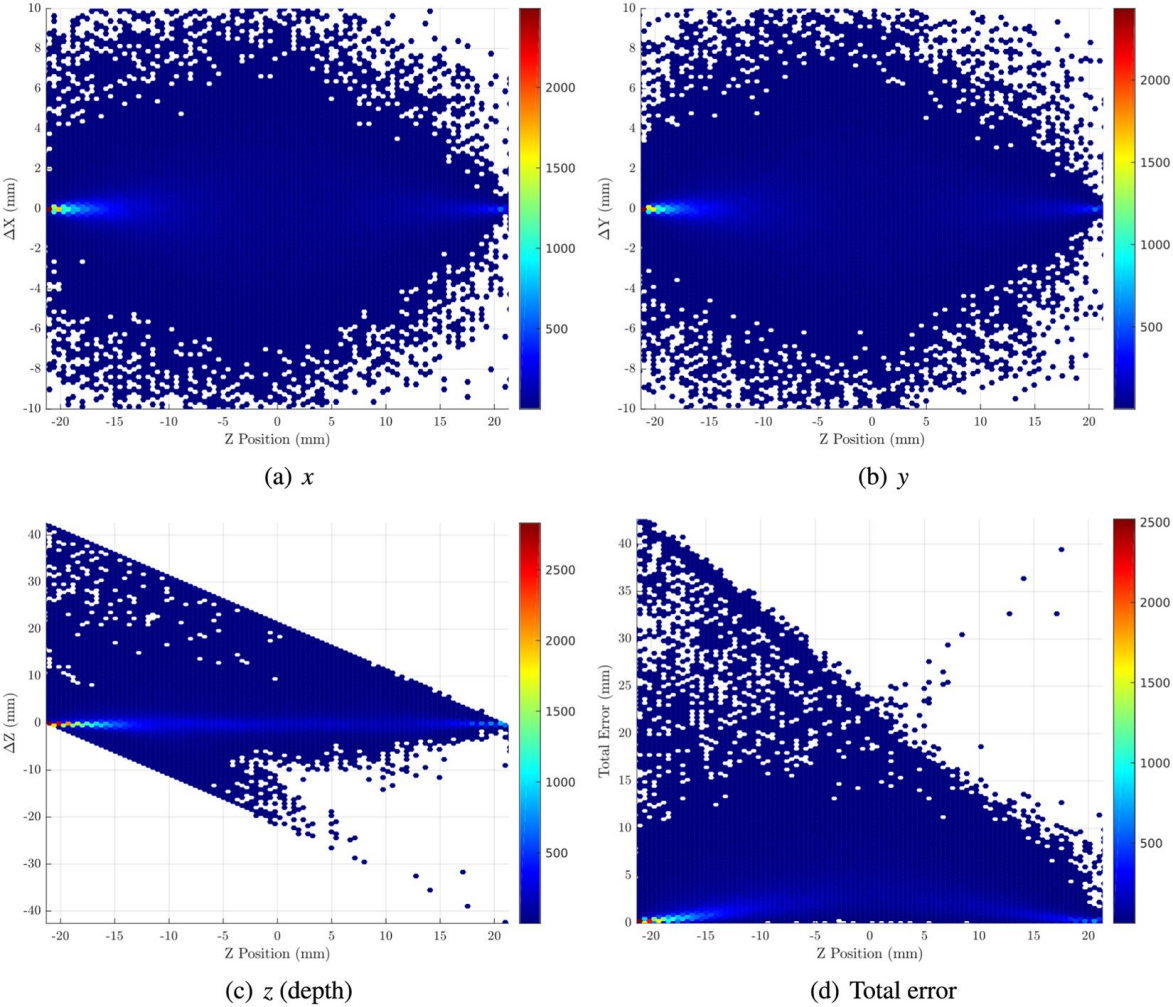
Distributions of error in position estimation in each dimension (for 200000 511 keV primary photons) as a function of depth for the optimal thickness of LaF_3_:Ce-PS monolithic scintillation detector. Figure 6(d) shows the distribution of total Euclidian error. Figures prepared using Matlab 2019b^78^.

**Figure 7:**
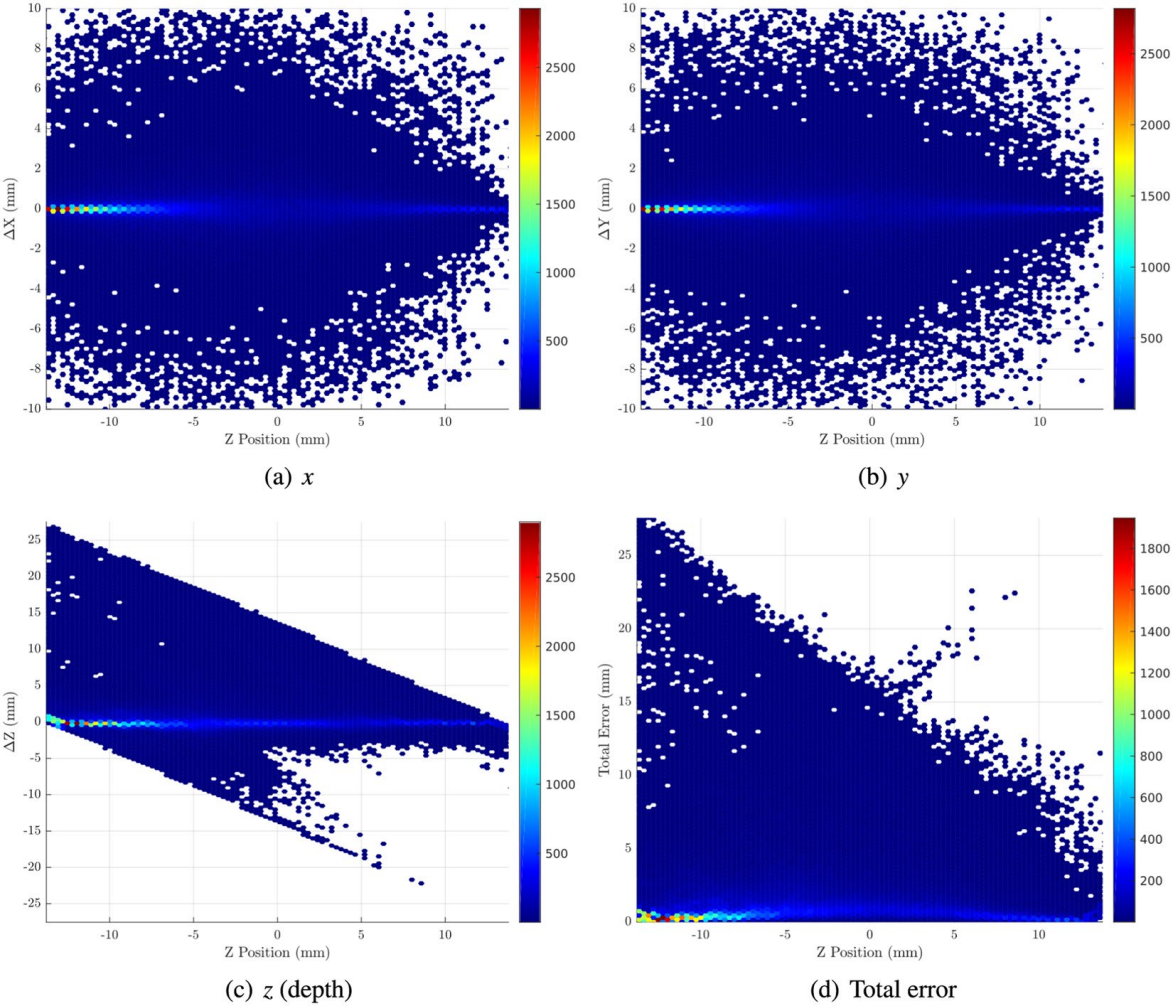
Distributions of error in position estimation in each dimension (for 200000 511 keV primary photons) as a function of depth for an optimal thickness of GLuGAG:Ce monolithic scintillation detector. Figure 7(d) shows the distribution of total Euclidian error. Figures prepared using Matlab 2019b^78^.

Figures 6 and 7 show a set of scatterplots showing position estimation distribution error in each dimension and in total as a function of depth for optimised slabs of LaF_3_:Ce-PS and GLuGAG:Ce, respectively. For these materials, the majority of errors in each dimension are within <±1 mm, with the outliers mostly corresponding to multi-interaction events. The greatest lateral error (*x* and *y*) occurs around the half-thickness of the scintillator slab, since this point is the furthest from either front or back detector and therefore most susceptible to scatter and attenuation. The higher density of the ceramic results in a larger fraction of events being detected close to the front surface compared to the nanocomposite, resulting in an overall superior performance for this material. These general observations also apply to the other nanocomposite and ceramic scintillators.

## Conclusion

A general method for optimising the thickness of a monolithic scintillator detector via Monte Carlo simulations was developed and demonstrated with a range of nanocomposite and transparent ceramic scintillator materials. The optimisation criterion which is maximised is the probability of detecting and localising the position of gamma interactions with the scintillator volume to within a specified margin of error. The method enables the optimisation process to be tuned according to the required minimum acceptable localisation accuracy, and can be adapted to a variety of monolithic scintillator localisation techniques beyond those discussed in this work. Importantly, this work demonstrates that for monolithic scintillator materials, determination of ‘optimal thickness’ is more complex than considering only the linear attenuation coefficient of the scintillator material.

Of the evaluated materials, the most promising nanocomposite scintillator for PET is LaF_3_:Ce-polystyrene, which is largely due the high loading factor which can be achieved without compromising its optical transparency at the scintillation emission wavelength. The best-performing ceramic scintillator was GLuGAG:Ce due to its higher optical transmittivity relative to the other evaluated ceramics; however, due to their high density, the ceramic scintillators all offered a higher achievable sensitivity compared to the nanocomposites. Each of these materials could potentially be used in PET applications, however the trade-off between sensitivity and the accuracy in determining the endpoints of the lines of response is different in each case and must also be balanced against the cost of each material.

The developed technique can be extended to any new nanocomposite or ceramic scintillator material, provided the physical and optical properties can be accurately measured.

## Supplementary Information

Supplementary Information.

## Data Availability

All data generated or analysed during this study are included in this published article (and its Supplementary Information files) or are available from the corresponding author on reasonable request. All source code is available via the following URL: https://bitbucket.org/keenanwilson/nanocomposite_ceramic_thickness_optimisation.git.
