# Supplementary material for: Optimisation of monolithic nanocomposite and transparent ceramic scintillation detectors for positron emission tomography
Source: Sci Rep. 2020 Jan 29;10:1409. doi: 10.1038/s41598-020-58208-y (PMC6989685; doi:10.1038/s41598-020-58208-y)
Supplement: Supplementary file 1 — Supplementary Information. [file 41598_2020_58208_MOESM1_ESM.pdf]

Optimisation of monolithic nanocomposite and transparent ceramic scintillation detectors for positron emission tomography

Keenan J. Wilson[1], Roumani Alabd[1], Mehran Abolhasan[1], Mitra Safavi-Naeini[2], and Daniel R. Franklin[1,*]

[1]School of Electrical and Data Engineering, University of Technology Sydney, NSW, Australia
[2]Australian Nuclear Science and Technology Organisation (ANSTO), NSW, Australia
[*]Daniel.Franklin@uts.edu.au

1 Optimisation of nanocomposite loading

Supplementary Figure 1(a) and 1(b) demonstrate that for nanocomposite materials, optical transmittivity and stopping power are both strongly influenced by the scintillator loading fraction; however, the effects are in opposition to one another. Higher loading (i.e. a higher fraction of nanoscintillator compared to the matrix material) results in a higher average density and effective atomic number (and hence stopping power); however, optical attenuation is increased, limiting the benefits of a high loading factor since many of the scintillation photons are scattered and/or absorbed before they can be detected.

To ensure that enough scintillation photons can be detected for an accurate estimate of the point of interaction to be determined, a reasonable approach to choosing an appropriate loading factor is to balance the optical attenuation length equally against the gamma radiation attenuation length. If a DSR detector configuration is being used, with this approach, the thickness of the scintillator which satisfies this criterion is doubled compared to a single-sided readout design. The scintillator loading volume fraction may be found by considering Supplementary Figure 1(a). For example, a 2 cm thick nanocomposite detector made from LaF_3:Ce nanoscintillator material in an oleic acid matrix (with double-sided readout) would require a 1 cm optical attenuation length to satisfy the condition, corresponding to approximately 17% scintillator loading by volume.

Alternatively, if a specific attenuation length is required - for example, 50% attenuation of 511 keV gamma photons (the half value layer or HVL) - then a suitable loading factor and thickness can be computed such that the gamma radiation attenuation length equals the optical attenuation length. In the case of a single-sided detector, the point of intersection of the curves in Supplementary Figure 1(a) and 1(b) can be found; for the DSR configuration, the optical attenuation length only needs to be equal to half of the HVL. This may be more easily seen in figure 2, which shows both of these plots overlaid for LaF_3:Ce OA. The scintillator volume fraction satisfying the condition in this case is $\sim$5%, at the point of intersection.

A summary of the loading factors used in the main body of work are shown in Supplementary Table 1.

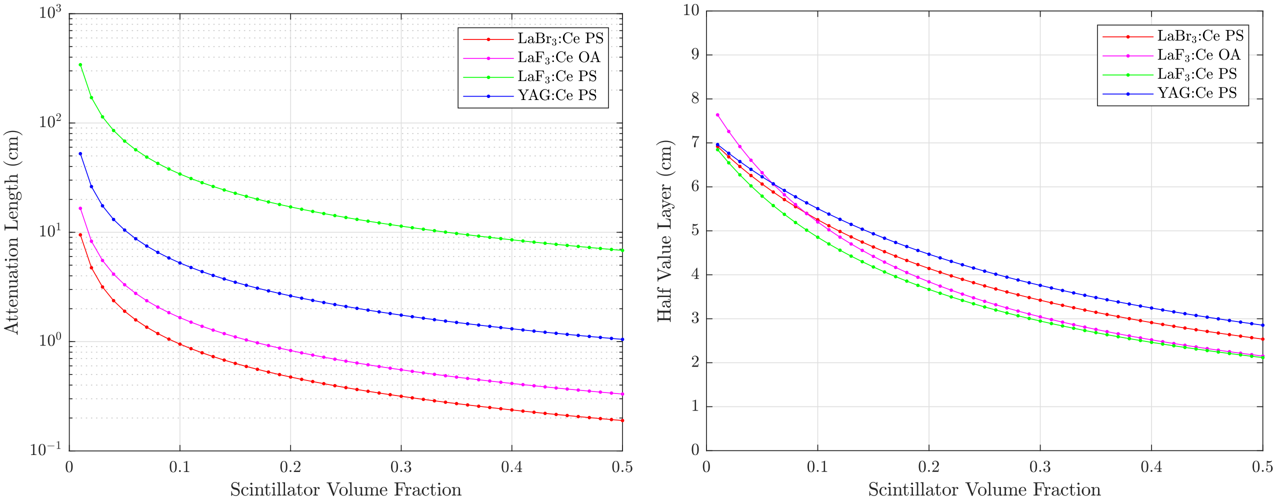

(a) Optical attenuation length (b) γ half-value layer thickness

Supplementary Figure 1. Theoretical optical attenuation length due to Rayleigh scatter, and half-value layer thickness (thickness at which 511 keV gamma attenuation is 50%), both expressed as a function of scintillator loading factor. All nanoparticles are assumed to be spherical and uniformly distributed, with a constant diameter of 9 nm (for comparison purposes). Material properties are based on data from the National Institute of Standards and Technology[1].

Supplementary Table 1. A summary of the loading factors by % volume, used in these simulations. Calculated as described, using figures 1(a) and 1(b). The loading factor for optimal thickness (denoted T. Opt.) was chosen based on a 3 cm thick slab, using the same methodology.

**Nanoparticle Matrix**	$LaBr_3$:Ce PS	Gd_2O_3 PVT	LaF_3:Ce OA	LaF_3:Ce PS	YAG:Ce PS
1 cm load (%)	19	4.6	34	50	50
2 cm Load (%)	9	4.6	17	50	50
HVL Load (%)	3	4.6	5	50	28
T. Opt. (%)	7	4.6	12	50	37

2 Impact of loading factor on optimal thickness

For further exploration of the effects of nanoparticle loading factor, a number of optimal thickness calculations were completed for a range of different loading factors. Supplementary Figure 3(a), 3(b), 3(c) and 3(d) show the optimal thickness of LaF_3:Ce OA for 5%, 12%, 20% and 30% scintillator loading respectively. Supplementary Figure 4(a), 4(b), 4(c) and 4(d) show the optimal thickness of LaF_3:Ce PS for 20%, 40%, 50% and 60% scintillator loading respectively. The optimal thickness and probability of detection for LaF_3:Ce OA and LaF_3:Ce PS are summarised in Supplementary Table 2 and 3, respectively. Each simulation consisted of 10000 primary events.

Supplementary Table 2. Optimum scintillator thickness (denoted T. Opt.) and corresponding probability of detection (P. D.) within a limit of 1, 2, 3, 4, 5 and ∞ mm. ΔD is the total error in position estimation for the point of interaction, using LaF_3:Ce oleic acid.

Loading (%)	$\Delta D \leq 1$ mm		$\Delta D \leq 2$ mm		$\Delta D \leq 3$ mm		$\Delta D \leq 4$ mm		$\Delta D \leq 5$ mm		All Detections	
	T. Opt. (mm)	P. D. (%)	T. Opt. (mm)	P. D. (%)	T. Opt. (mm)	P. D. (%)	T. Opt. (mm)	P. D. (%)	T. Opt. (mm)	P. D. (%)	T. Opt. (mm)	P. D. (%)
5	21.56	12.34	28.71	17.73	34.99	20.87	39.37	23.39	41.34	24.97	62.64	39.77
12	19.58	13.58	24.26	19.33	28.50	22.15	30.78	24.58	32.66	25.73	39.42	33.97
20	14.60	14.88	20.85	20.77	23.91	23.59	25.66	25.23	26.48	26.49	29.25	32.97
30	13.21	16.53	16.65	21.86	17.71	24.24	18.26	25.43	18.56	26.26	21.10	31.32

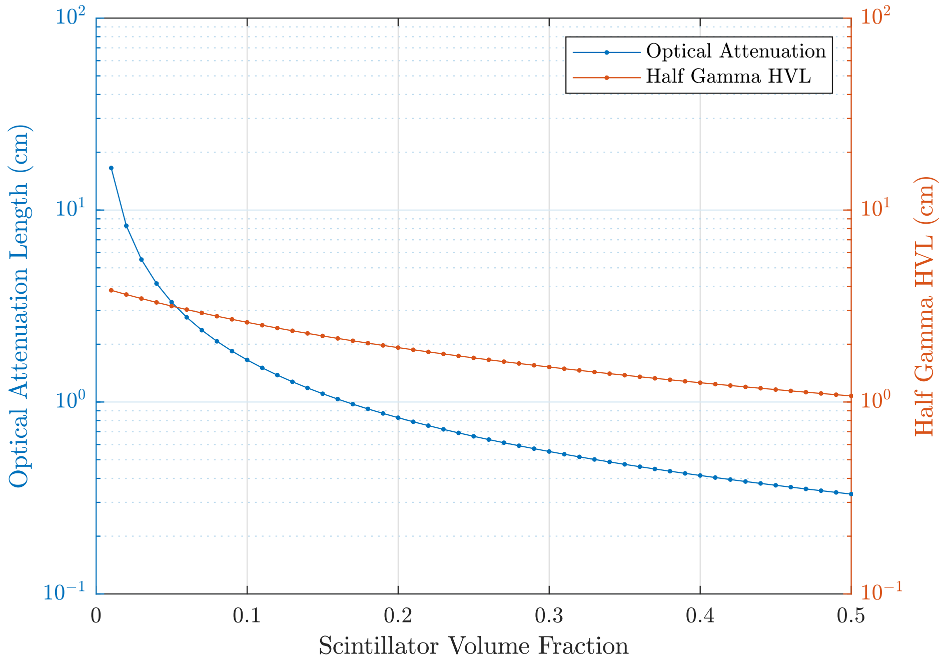

Supplementary Figure 2. Overlaid plots of optical attenuation and HVL / 2 for LaF_3:Ce oleic acid. The point of intersection between these curves is used to determine appropriate scintillator loading factor for the nanocomposite; in this case, the point of intersection occurs at ∼5%.

Supplementary Table 3. Optimum scintillator thickness (denoted T. Opt.) and corresponding probability of detection (P. D.) within a limit of 1, 2, 3, 4, 5 and ∞ mm. ΔD is the total error in position estimation for the point of interaction, using LaF_3:Ce polystyrene.

Loading (%)	ΔD ≤ 1 mm		ΔD ≤ 2 mm		ΔD ≤ 3 mm		ΔD ≤ 4 mm		ΔD ≤ 5 mm		All Detections	
	T. Opt. (mm)	P. D. (%)	T. Opt. (mm)	P. D. (%)	T. Opt. (mm)	P. D. (%)	T. Opt. (mm)	P. D. (%)	T. Opt. (mm)	P. D. (%)	T. Opt. (mm)	P. D. (%)
20	20.01	18.54	25.48	25.31	39.00	29.91	41.40	32.84	42.88	34.88	-	-
40	19.63	24.61	26.38	33.49	31.83	37.99	37.92	41.42	45.83	44.40	-	-
50	19.21	26.23	26.61	35.73	36.35	40.95	39.41	45.66	42.60	48.83	-	-
60	19.55	28.29	27.00	38.71	31.67	44.65	37.13	48.90	44.25	52.37	65.86	83.16

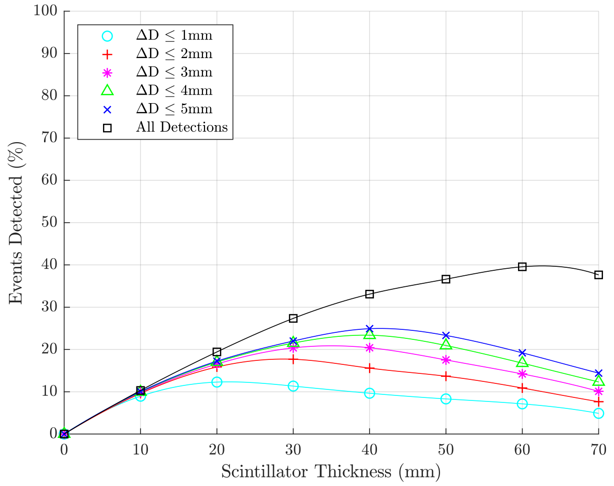

(a) LaF_3:Ce OA (5 % loading)

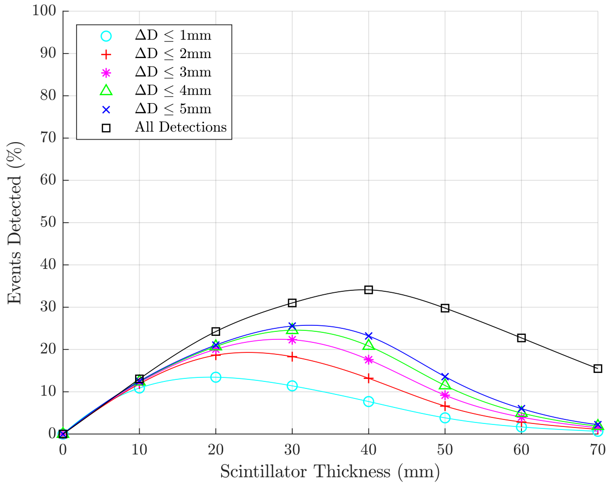

(b) LaF_3:Ce OA (12 % loading)

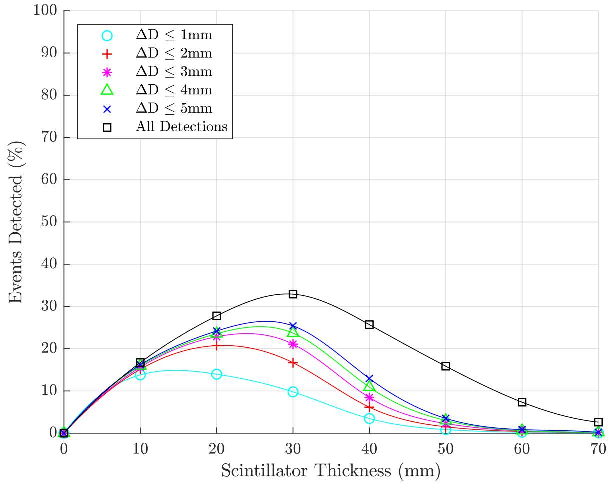

(c) LaF_3:Ce OA (20 % loading)

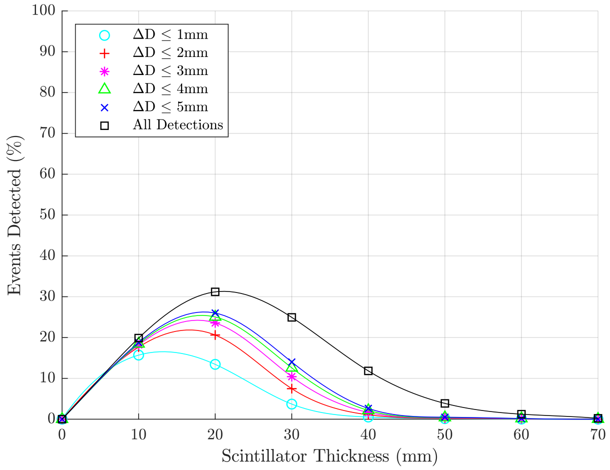

(d) LaF_3:Ce OA (30 % loading)

Supplementary Figure 3. Percentage of events detected to a specified accuracy of 1 mm, 2 mm, 3 mm, 4 mm, 5 mm and all detections as a function of scintillator thicknesses for LaF_3:Ce oleic acid.

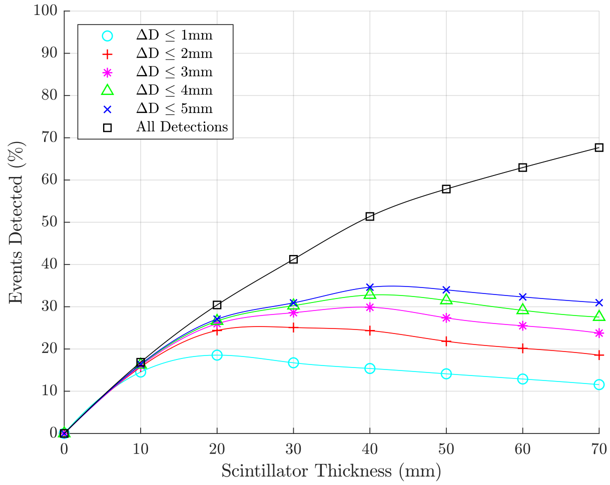

(a) LaF_3:Ce PS (20 % loading)

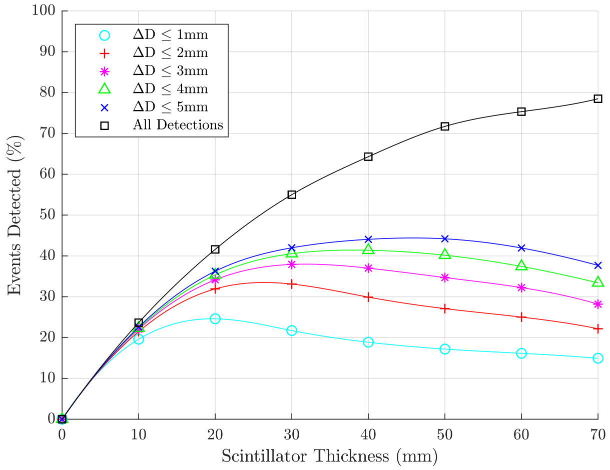

(b) LaF_3:Ce PS (40 % loading)

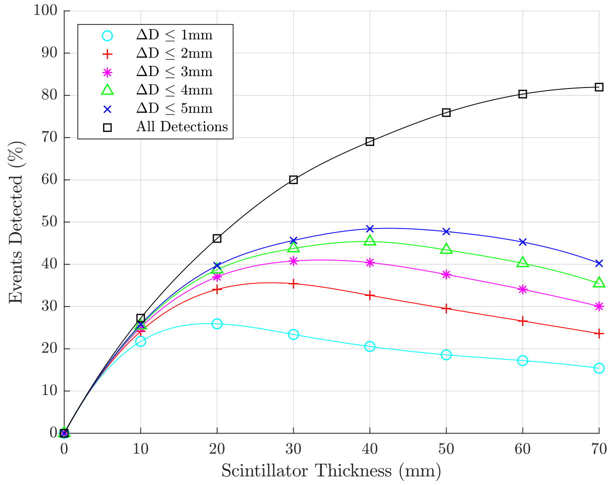

(c) LaF_3:Ce PS (50 % loading)

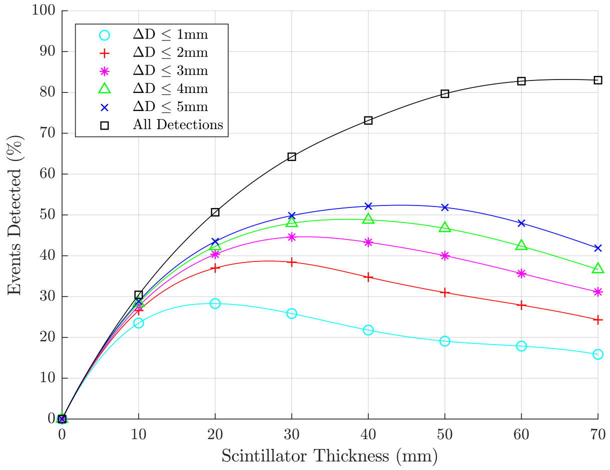

(d) LaF_3:Ce PS (60 % loading)

Supplementary Figure 4. Percentage of events detected to a specified accuracy of 1 mm, 2 mm, 3 mm, 4 mm, 5 mm and all detections as a function of scintillator thicknesses for LaF_3:Ce polystyrene.

3 Analysis of photoelectric vs. Compton interactions

The probability of photoelectric, single and multiple Compton interactions in each scintillator type is shown in Tables 4, 5 and 6 for 1 cm, 2 cm and half-value layer thickness monolithic slabs. LSO has been used as a benchmark scintillator for comparison with these materials, as may be seen in Tables 4, 5 and 6, which show the percentage and composition of events detected for scintillators of 1 cm, 2 cm and the HVL of each material, respectively.

The analytic function used for estimating the location of the point of interaction assumes that all interactions are photoelectric. In reality, many interactions involve one or more Compton (incoherent) scatter events, which potentially results in more than one point of energy deposition within the scintillator slab. Since energy is lost in each Compton interaction, the probability of photoelectric absorption increases with each subsequent scatter. The result is a more spread-out (and, in general, irregular) projection of optical photons on the detector surface following a multi-interaction event compared to a simple photoelectric interaction or a Compton interaction where the scattered photon does not undergo any further interaction with the scintillator.

The cross-sections determining the probability of interaction via photoelectric absorption and Compton scattering are complex functions of the electronic structure of the atoms in the photon path. Photoelectric absorption cross-section is approximately proportional to the 4th or 5th power of Z_{eff} and proportional to the 3.5th power of wavelength. By contrast, Compton cross-section is approximately proportional to the density of the material and wavelength. At 511 keV, the dominant process is Compton scattering for both nanocomposite and ceramic scintillators, although a substantial minority of photons are absorbed photoelectrically in both cases (much more so for the ceramics). The majority of cases for the evaluated scintillator materials listed in Tables 4-6 involve either purely photoelectric interactions, single or double Compton scatter events (more heavily weighted towards photoelectric and single-Compton events in the case of the ceramic materials).

The high loading factor of LaF_3:Ce-PS makes it the best-performing nanocomposite in terms of the proportion of photoelectric interactions. All of the transparent ceramic materials offer performance which is much more similar to the benchmark performance of the LSO than even the LaF_3:Ce-PS nanocomposite, both in terms of the total proportion of events detected and the percentage of these interactions which are photoelectric. In terms of scattering composition, LuAG:Pr has characteristics most similar to the LSO with only $\sim$4% difference in the number of photoelectric interactions recorded, though GLuGAG:Ce and GAGG:Ce still have respectable numbers ($\sim$10% and 13% difference respectively). GYGAG:Ce has less then half the photoelectric count of LSO, again due to a lower density. Interestingly, LuAG:Pr has the highest percentage of events detected at 1 cm scintillator thickness, but as this is increased to 2 cm all other ceramics have a higher rate of increase (with GLuGAG:Ce increasing the most).

Multi-interaction events degrade the accuracy of the fitting algorithm, which assumes all energy is deposited at a single location. Fortunately, since the fraction of photon energy deposited is related to the angle of scatter, the magnitude of this degradation is smaller than at first may appear. If the first interaction deposits a large fraction of the gamma photon's energy, the angle of scatter will also be large - but the reduction in photon energy reduces the mean free path length of the scattered photon compared to the initial 511 keV gamma photon. Therefore, the resulting optical photon distribution will be dominated by the first point of interaction, with only a minor impact on the position of the endpoint of the line of response. If the amount of energy deposited at the first point of interaction is small, the angle of scatter will also be small. Therefore, subsequent interactions will follow a trajectory through the scintillator which is not very different to that of the original photon. If the next interaction is photoelectric (which is now more likely as the photon energy has been reduced), the resulting line of response will only deviate

Supplementary Table 4. Distribution of photoelectric and Compton-scattered interactions for a 1 cm thick scintillator slab, expressed as the percentage of events of this type which are detected out of the total number of primary photons incident on the detector. C*N* denotes *N* Compton scatter interactions prior to either final photoelectric absorption or escape from the scintillator. Monocrystalline LSO is also included as a reference benchmark.

**Scintillator**	**Photoelectric/Compton scatter composition**						**Total events detected** (%)
	PE (%)	C1 (%)	C2 (%)	C3 (%)	C4 (%)	C5+ (%)	
Gd_2O_3 PVT	1.58	87.98	9.37	0.91	0.14	0.02	11.70
$LaBr_3$:Ce PS	2.47	84.04	11.63	1.62	0.22	0.02	15.17
LaF_3:Ce OA	6.55	76.00	14.46	2.51	0.41	0.07	20.81
LaF_3:Ce PS	9.75	71.17	15.41	3.05	0.56	0.07	27.02
YAG:Ce PS	1.89	78.67	15.47	3.23	0.62	0.12	21.68
GAGG:Ce	26.95	53.13	15.73	3.51	0.57	0.11	45.12
GLuGAG:Ce	24.34	55.14	16.33	3.55	0.57	0.07	48.50
GYGAG:Ce	14.08	61.98	18.33	4.57	0.86	0.17	40.14
LuAG:Pr	30.09	52.47	14.16	2.84	0.39	0.04	49.79
LSO	34.85	49.36	13.11	2.34	0.31	0.03	55.57

Supplementary Table 5. Distribution of photoelectric and Compton-scattered interactions for a 2 cm thick scintillator slab.

**Scintillator**	**Photoelectric/Compton scatter composition**						**Total events detected** (%)
	PE (%)	C1 (%)	C2 (%)	C3 (%)	C4 (%)	C5+ (%)	
Gd_2O_3 PVT	1.34	83.90	12.56	1.90	0.22	0.07	21.29
$LaBr_3$:Ce PS	1.11	83.99	12.72	1.84	0.28	0.06	22.08
LaF_3:Ce OA	3.32	77.77	15.57	2.76	0.49	0.10	26.09
LaF_3:Ce PS	9.84	64.15	19.87	4.96	0.95	0.23	45.09
YAG:Ce PS	1.86	71.85	19.76	4.95	1.21	0.37	37.82
GAGG:Ce	30.41	41.14	20.79	6.15	1.28	0.23	41.18
GLuGAG:Ce	24.33	49.12	19.88	5.39	1.06	0.21	71.59
GYGAG:Ce	14.14	54.38	22.75	6.84	1.56	0.34	62.65
LuAG:Pr	31.20	45.98	17.76	4.18	0.77	0.10	67.50
LSO	34.86	45.00	15.95	3.53	0.58	0.08	78.85

Supplementary Table 6. Distribution of photoelectric and Compton-scattered interactions for a scintillator thickness equal to the half value layer for each scintillator material.

Scintillator	Photoelectric/Compton scatter composition						Total events detected (%)	Thickness HVL (mm)
	PE (%)	C1 (%)	C2 (%)	C3 (%)	C4 (%)	C5+ (%)		
Gd_2O_3 PVT	0.92	77.69	17.49	3.19	0.60	0.12	39.56	53.10
$LaBr_3$:Ce PS	0.14	79.93	16.77	2.65	0.43	0.08	40.30	64.40
LaF_3:Ce OA	0.28	81.42	15.45	2.47	0.33	0.05	26.92	64.90
LaF_3:Ce PS	10.06	63.76	19.99	4.98	1.01	0.21	46.25	21.20
YAG:Ce PS	1.11	71.55	20.98	4.92	1.10	0.33	43.26	39.50
GAGG:Ce	26.91	53.04	15.80	3.52	0.64	0.08	46.64	10.60
GLuGAG:Ce	24.34	55.14	16.33	3.55	0.57	0.07	48.50	10.00
GYGAG:Ce	14.39	58.90	20.06	5.30	1.11	0.24	48.52	13.10
LuAG:Pr	30.13	52.73	14.02	2.74	0.34	0.04	47.30	9.29
LSO	35.14	50.59	12.04	1.96	0.26	0.02	48.10	7.99

Supplementary Table 7. Distribution of photoelectric and Compton-scattered interactions for a scintillator thickness equal to the optimal thickness for each scintillator material.

Scintillator	Photoelectric/Compton scatter composition						Total events detected (%)	Thickness Opt. (mm)
	PE (%)	C1 (%)	C2 (%)	C3 (%)	C4 (%)	C5+ (%)		
Gd_2O_3 PVT	0.79	76.72	18.51	3.24	0.60	0.14	43.23	62.61
$LaBr_3$:Ce PS	0.32	79.76	16.65	2.71	0.45	0.10	40.57	53.78
LaF_3:Ce OA	1.20	79.75	15.83	2.64	0.48	0.09	30.84	32.66
LaF_3:Ce PS	11.51	55.73	23.62	7.08	1.69	0.38	60.21	42.60
YAG:Ce PS	1.64	68.30	22.42	5.76	1.42	0.46	50.43	49.79
GAGG:Ce	22.17	52.64	19.34	4.81	0.90	0.14	53.03	13.98
GLuGAG:Ce	25.15	46.13	21.07	6.17	1.23	0.24	78.38	27.54
GYGAG:Ce	15.30	47.61	25.34	8.83	2.36	0.56	79.72	42.63
LuAG:Pr	31.61	46.25	17.24	4.08	0.71	0.11	67.61	18.96

Supplementary Table 8. Mean and median errors in the estimation of the point of interaction within a 1 cm thick scintillator slab, in each dimension and overall. Standard deviations and interquartile ranges (the spread of the middle 50% of errors) are also listed.

Scintillator	x error (mm)				y error (mm)				z error (mm)				Total error (mm)			
	Med.	Mean	IQR	SD	Med.	Mean	IQR	SD	Med.	Mean	IQR	SD	Med.	Mean	IQR	SD
Gd_2O_3 PVT	1×10^{-5}	4×10^{-3}	0.1	0.5	-3×10^{-4}	-3×10^{-3}	0.1	0.5	-1×10^{-1}	-6×10^{-2}	0.2	0.7	0.2	0.4	0.2	0.9
$LaBr_3$:Ce PS	-5×10^{-4}	-7×10^{-4}	0.1	0.6	-3×10^{-4}	-3×10^{-4}	0.1	0.6	-1×10^{-1}	-4×10^{-2}	0.1	0.9	0.2	0.5	0.2	1.2
LaF_3:Ce OA	1×10^{-3}	3×10^{-3}	0.3	1.0	-2×10^{-3}	-6×10^{-4}	0.3	1.0	-1×10^{-1}	6×10^{-2}	0.4	1.3	0.5	1.0	0.6	1.6
LaF_3:Ce PS	-6×10^{-4}	-4×10^{-3}	0.3	1.0	8×10^{-4}	3×10^{-3}	0.3	1.0	-1×10^{-1}	3×10^{-2}	0.3	1.2	0.4	0.9	0.5	1.6
YAG:Ce PS	-4×10^{-4}	-6×10^{-3}	0.1	0.6	4×10^{-5}	-2×10^{-3}	0.1	0.6	-1×10^{-1}	6×10^{-3}	0.4	1.0	0.3	0.6	0.3	1.2
GAGG:Ce	2×10^{-5}	-4×10^{-3}	0.2	1.2	-3×10^{-4}	-2×10^{-3}	0.2	1.2	-1×10^{-1}	1×10^{-1}	0.4	1.4	0.3	1.0	0.6	1.9
GLuGAG:Ce	-2×10^{-4}	-2×10^{-3}	0.1	1.1	-4×10^{-4}	2×10^{-3}	0.1	1.1	-1×10^{-1}	2×10^{-1}	0.4	1.4	0.3	1.0	0.6	1.8
GYGAG:Ce	-7×10^{-5}	1×10^{-3}	0.1	1.1	-8×10^{-5}	1×10^{-3}	0.1	1.0	-1×10^{-1}	1×10^{-1}	0.3	1.3	0.3	0.9	0.5	1.8
LuAG:Pr	2×10^{-5}	4×10^{-3}	0.2	1.1	-5×10^{-4}	4×10^{-4}	0.2	1.1	-1×10^{-1}	2×10^{-1}	0.5	1.4	0.4	1.1	0.7	1.8
LSO	-3×10^{-4}	-7×10^{-3}	0.1	1.1	9×10^{-5}	1×10^{-3}	0.1	1.1	-1×10^{-1}	3×10^{-1}	0.4	1.4	0.3	1.0	0.7	1.8

Supplementary Table 9. Mean and median errors in the estimation of the point of interaction within a 2 cm thick scintillator slab, in each dimension and overall. Standard deviations and interquartile ranges (the spread of the middle 50% of errors) are also listed.

Scintillator	x error (mm)				y error (mm)				z error (mm)				Total error (mm)			
	Med.	Mean	IQR	SD	Med.	Mean	IQR	SD	Med.	Mean	IQR	SD	Med.	Mean	IQR	SD
Gd_2O_3 PVT	4×10^{-4}	4×10^{-3}	0.3	0.8	-4×10^{-4}	-6×10^{-3}	0.3	0.8	-1×10^{-1}	0.3	0.3	2.3	0.4	1.0	0.4	2.4
$LaBr_3$:Ce PS	-2×10^{-4}	1×10^{-3}	0.2	0.8	-1×10^{-4}	-7×10^{-4}	0.2	0.8	-1×10^{-1}	0.2	0.2	1.9	0.3	0.8	0.3	2.1
LaF_3:Ce OA	-2×10^{-4}	-7×10^{-3}	0.6	1.4	2×10^{-3}	-2×10^{-3}	0.6	1.4	-1×10^{-1}	0.5	0.8	2.8	0.9	1.8	1.3	2.9
LaF_3:Ce PS	-1×10^{-4}	2×10^{-3}	0.6	1.4	-2×10^{-5}	-2×10^{-3}	0.6	1.4	-9×10^{-2}	0.6	0.7	3.0	0.8	1.9	1.4	3.1
YAG:Ce PS	1×10^{-5}	4×10^{-3}	0.3	1.0	-1×10^{-3}	-1×10^{-3}	0.3	1.0	-1×10^{-1}	0.4	0.9	2.4	0.6	1.4	1.0	2.5
GAGG:Ce	6×10^{-4}	1×10^{-2}	0.8	1.9	9×10^{-4}	1×10^{-2}	0.8	1.9	-2×10^{-2}	0.8	1.4	3.4	1.2	2.7	2.7	3.5
GLuGAG:Ce	6×10^{-5}	-4×10^{-3}	0.4	1.5	-3×10^{-4}	-4×10^{-3}	0.4	1.5	-9×10^{-2}	0.7	0.8	2.8	0.6	1.9	1.6	3.1
GYGAG:Ce	2×10^{-4}	3×10^{-3}	0.3	1.5	-1×10^{-4}	-4×10^{-3}	0.3	1.5	-1×10^{-1}	0.7	0.6	2.9	0.4	1.8	1.6	3.2
LuAG:Pr	-7×10^{-4}	-3×10^{-3}	0.5	1.5	-4×10^{-4}	3×10^{-4}	0.5	1.5	-6×10^{-2}	0.7	1.0	2.7	0.8	2.0	1.6	2.9
LSO	1×10^{-4}	2×10^{-3}	0.3	1.4	2×10^{-4}	-2×10^{-3}	0.3	1.4	-9×10^{-2}	0.9	0.8	2.8	0.4	1.8	1.6	3.0

slightly from the true line of response (and will be slightly pulled back towards the true LoR by whatever energy was deposited at the first point of interaction). The largest error introduced by such events will be in the radial direction (depth). The intermediate case - an initial or secondary Compton scatter which deposits some non-trivial amount of energy, followed by further multiple Compton interactions, results in the largest errors in the placement of the line of response.

4 Position error estimates - fixed thickness scintillator

This section presents several additional results comparing the distributions of localisation error for fixed scintillator slab thicknesses of 1 cm (Supplementary Table 8) and 2 cm (Supplementary Table 9).

Supplementary Table 10. Mean and median errors in the estimation of the point of interaction within a scintillator slab with thickness equal to the half-value layer (HVL), in each dimension and overall. Standard deviations and interquartile ranges (the spread of the middle 50% of errors) are also listed.

Scintillator	x error (mm)				y error (mm)				z error (mm)				Total error (mm)			
	Med.	Mean	IQR	SD	Med.	Mean	IQR	SD	Med.	Mean	IQR	SD	Med.	Mean	IQR	SD
Gd_2O_3 PVT	-1×10^{-3}	-2×10^{-3}	0.8	1.7	2×10^{-3}	1×10^{-2}	0.8	1.7	-1×10^{-1}	1.3	0.9	6.8	1.2	2.9	2.1	6.7
$LaBr_3$:Ce PS	4×10^{-4}	2×10^{-3}	0.7	1.7	5×10^{-4}	-2×10^{-3}	0.7	1.7	-1×10^{-1}	0.5	0.9	4.1	1.2	2.3	2.3	4.2
LaF_3:Ce OA	4×10^{-3}	6×10^{-3}	2.3	3.3	3×10^{-4}	4×10^{-3}	2.4	3.3	-4×10^{-2}	1.7	4.0	9.3	4.3	6.8	7.1	8.1
LaF_3:Ce PS	-1×10^{-3}	5×10^{-4}	0.6	1.5	-2×10^{-4}	-6×10^{-3}	0.6	1.5	-1×10^{-1}	0.7	0.8	3.3	0.8	2.1	1.5	3.4
YAG:Ce PS	-7×10^{-4}	-2×10^{-3}	0.6	1.4	-8×10^{-4}	-2×10^{-3}	0.6	1.4	-1×10^{-1}	0.6	0.8	4.0	0.9	2.0	1.4	4.0
GAGG:Ce	-2×10^{-6}	5×10^{-4}	0.2	1.2	-2×10^{-4}	-2×10^{-4}	0.2	1.2	-1×10^{-1}	0.2	0.4	1.5	0.3	1.1	0.7	1.9
GLuGAG:Ce	-2×10^{-4}	-2×10^{-3}	0.1	1.1	-4×10^{-4}	2×10^{-3}	0.1	1.1	-1×10^{-1}	0.2	0.4	1.4	0.3	1.0	0.6	1.8
GYGAG:Ce	-1×10^{-4}	-3×10^{-4}	0.2	1.2	2×10^{-4}	-6×10^{-3}	0.2	1.2	-1×10^{-1}	0.3	0.4	1.8	0.3	1.2	0.8	2.2
LuAG:Pr	5×10^{-4}	2×10^{-3}	0.2	1.0	-3×10^{-4}	1×10^{-3}	0.2	1.0	-1×10^{-1}	0.1	0.5	1.3	0.4	1.0	0.6	1.7
LSO	3×10^{-4}	3×10^{-3}	0.1	1.0	-1×10^{-4}	-8×10^{-4}	0.1	1.0	-1×10^{-1}	0.1	0.3	1.1	0.3	0.9	0.5	1.5

5 Position error estimates - half value layer (50% photon attenuation) scintillator

This section presents the distributions of localisation error for scintillator slabs with thicknesses such that 50% of incident photons are absorbed (half-value layer thickness) (Supplementary Table 10).

References

1. Berger, M. *et al.* XCOM: Photon Cross Section Database (version 1.5) (2010). URL https://physics.nist.gov/PhysRefData/Xcom/html/xcom1.html.
